# Veterinary Education and Training on Non-Traditional Companion Animals, Exotic, Zoo, and Wild Animals: Concepts Review and Challenging Perspective on Zoological Medicine

**DOI:** 10.3390/vetsci10050357

**Published:** 2023-05-17

**Authors:** Jaime Espinosa García-San Román, Óscar Quesada-Canales, Manuel Arbelo Hernández, Soraya Déniz Suárez, Ayoze Castro-Alonso

**Affiliations:** 1 Department of Animal Pathology, Veterinary School, University of Las Palmas de Gran Canaria (ULPGC), 35413 Arucas, Spain; 2 Veterinary Histology and Pathology, Institute of Animal Health and Food Safety (IUSA), University of Las Palmas de Gran Canaria (ULPGC), 35413 Arucas, Spain; oscar.quesada@ulpgc.es (Ó.Q.-C.);; 3 Veterinary Infectious Diseases and Ichthyopathology, Veterinary School, University of Las Palmas de Gran Canaria (ULPGC), 35413 Arucas, Spain

**Keywords:** zoological medicine, exotic animals, veterinary, One Health, education, NTCAs, companion animals

## Abstract

**Simple Summary:**

In the recent past, the concepts of exotic animal medicine and zoological medicine have been used wrongly or interchangeably. The veterinary community should adopt zoological medicine as the appropriate term to cover the veterinary medicine of non-traditional companion animals, zoo animals, and wildlife animals. Furthermore, zoological medicine should be also integrated into the ecosystem health evaluation (environmental medicine) and the “One Health” approachBeyond the terminology, zoological medicine has evolved and expanded its activities and action fields from its historical roots to afford new and challenging scenarios. In this context, the practice of zoological medicine includes its exercise in private veterinary clinics and hospitals, zoos and bioparks, and directly on wildlife. Several studies and international organisations have stressed the importance of high-quality veterinary training in this discipline, at the beginning and continuously during the professional practice. This fact is consistent with an increased societal demand for skilled and trained veterinarians concerning a broader species target and a wider scientific and technical scope including fields including eco-biology, environmental management, public health, welfare, and conservation. These challenging scenarios can only be correctly met by implementing more related compulsory subjects in the veterinary curriculum and post-degree specialisation and accreditation.

**Abstract:**

The role of veterinarians is becoming more significant and necessary to support the welfare and health not only of non-traditional companion animals and wildlife animals, but also of humans and the environment. The importance of the One Health/One World concept and its social impact is increasing significantly, accompanied by the notoriety of new emerging and reemerging zoonoses. This paper aims to review and anchor the main concepts and professional applications of zoological medicine, which has been extensively discussed and adapted in recent decades. In addition, we analyse the main social demands, training, and educational needs and the perception of veterinary professionals relating to this specialised veterinary discipline. Our final goal is to reinforce the use of the term zoological medicine and contribute to highlight the need to foster and underpin specific educational policies and programs on this matter in the veterinary curricula. Zoological medicine should be the appropriate and agreed-upon term in the academic language concerning the veterinary medicine of pets, wild, or zoo species, excluding traditional domestic animals, and integrating the principles of ecology and conservation, applied to both natural and artificial environments. This discipline has suffered an intense evolution covering applications in private clinics, zoos, bioparks, and wildlife. All this implies current and future challenges for the veterinary profession that can only be addressed with greater and better attention from multiple perspectives, especially the education and training of professionals to improve and specialise in their professional scope of services.

## 1. Introduction

Veterinary sciences have been concerned with animal health since ancient times (BAM 159 cuneiform tablet, Code of Hammurabi, Ugaritic text, Roman veterinary medicine). The focus has been established mostly on domestic animals, whether they are for production or companionship. In the last decades, the term “companion animals” has notably been extended from just traditional pets (cats, dogs, small birds, and rodents) to integrate new species such as exotic birds, rabbits, ferrets, snakes, lizards, and others which are legally covered to be kept in homes [1,2].

Human pressure on ecosystems and the continued loss of habitats and biodiversity have made necessary the implementation of important conservation and preservation measures where the role of veterinarians has progressively expanded and their contributions have increased in the last decades. Among these measures, it has been crucial to reorientate zoo policies from being menageries to becoming true centres of environmental education and ex situ conservation [3,4].

In addition, recent events, such as the SARS-CoV-2 pandemic or monkeypox, have highlighted the relevance of establishing robust health surveillance and monitoring programs for wild species populations and fostering the One Health/One World approach, that is, that human, animal, and environmental health are inextricably interconnected [5,6].

Veterinary science should no longer be focused exclusively on the maintenance of production or traditional domestic and companion animals. Instead, its scope of action is much broader, covering new species and scenarios [7]. These facts generate relevant challenges in adaptation and transformation in the veterinary profession, which needs to implement essential changes in educational and training programs to face these challenges and lead the job to a more open vision of health and welfare, not only animal, but also human and their environments.

This paper aims to review and anchor the main concepts and professional applications of zoological medicine, which has been extensively discussed and adapted in the last decades. In addition, we provide an analysis of main social demands, training, and educational needs and the perception of veterinary professionals relating to this specialised veterinary discipline. Our final goal is to reinforce the use of the term “zoological medicine” and contribute to highlight the need to foster and underpin specific educational policies and programs on this matter in the veterinary curricula.

## 2. Material and Methods

To produce this work, we have developed a scientific review, mainly at European and American scales, to consolidate past and current concepts of zoological medicine, to improve the understanding of the evolution of its scope and professional framework, and, finally, to contribute to update and define social demand and educational and training needs for this veterinary specialisation. In addition, to directly verify the implementation status of the clinical practice and professional perception of zoological medicine, a brief and anonymous survey has been conducted at the national level with the cooperation of regional veterinary professional associations in Spain.

To contribute to focusing on and clarifying the concept of zoological medicine, our analysis starts with the descriptions of the terms “one medicine” (1964) and “conservation medicine” (1996). We describe later the initial discussions of different authors at the beginning of the decade of 2000, mainly focused on the definition of the type of animals included under this concept, whether exotic or non-traditional companions animals. Finally, this review is expanded to the decades of 2010 and 2020, to integrate the importance of health applied to animals, humans, and the environment.

In this document, we understand that zoological medicine is a vast concept that encompasses different species and fields, including non-traditional companion animals, zoo animals, wildlife animals, aquatic animals, production and environmental medicines [1,7]. However, it could be really challenging to try to classify the professional application of zoological medicine based on the different species involved. Instead, our work affords this organisation based on the principal place to conduct veterinary services, including (i) private clinics, (ii) zoos and parks, and (iii) wildlife.

Lastly, this work analyses the social demands and educational needs of zoological medicine from the veterinary perspective. It includes reports based on direct studies and surveys to understand better the application of professional veterinary services to non-traditional companion animals and wild species in different parts of the world (Europe, Canada, USA, and South America). In addition, a brief national survey has been conducted among the professional veterinary associations in Spain (primarily private clinics) to reinforce and update these results. The survey was distributed on the internet and focused on obtaining data from veterinarians referring to their level of experience in zoological medicine, type of professional application, type of animals, main reasons for consultancy, their professional confidence, and training/educational needs. The participation in the survey was voluntary and anonymous and, before accessing the questions, all respondents agreed to and authorised the use of their answers for educational and research purposes. No personal data were collected, and all the information was aggregated for statistical purposes. This survey was outside of the scope of ethical concern and data protection law according to the EU General Data Protection Regulation (GDPR; Directive (EU) 2016/680).

## 3. Results and Discussion

### 3.1. Consolidating the Concepts and the Appropriate Terminology: Zoological Medicine and One Health

The terms “zoological medicine” and “exotic animal medicine” have often been used interchangeably, referring to animals other than those known as traditional domestic animals, i.e., dogs, cats, horses, or other farm animals [1,5]. However, these terms should be distinct. Zoological medicine has been defined as encompassing companion animal medicine (small mammals, birds, reptiles, amphibians, and fish), zoo animal medicine, aquatic animal medicine (marine mammals, display fish), production medicine (farmed/ranched wildlife, game birds, and aquaculture), and environmental medicine (free-ranging wildlife, conservation/preservation, and ecosystem health) [1,7].

In contrast, exotic animal medicine is commonly applied only to certain species of small mammals, reptiles, and birds kept as pets. The term “exotic” is defined in multiple veterinary dictionaries as relating to “an animal that is not indigenous to a location where it currently lives”. In this sense, a European rabbit could be native to mainland Spain but exotic in the United Kingdom, Australia, or the United States. In addition, as a small-prey herbivore species, the rabbit would behave much more like a wild animal than a domesticated dog [1].

In the European continent, the British Small Animal Veterinary Association (BSAVA) prefers the term “non-traditional companion animal” (NTCA) over the term “exotic pet” as it considers that this better describes the species involved [2]. In addition, it would be a mistake to infer that the diagnostic and treatment techniques used are markedly different if the animal is kept in a pet, zoo, or wildlife situation. They have more things in common for their approach to the taxon than based on the degree of domestication or the place of maintenance. For these reasons, it would be preferable to use the term zoological medicine for everything that covers the medicine of pets, wild or zoo species of reptiles, birds, and mammals excluded from traditional domestic ones such as dogs, cats, horses, and farm animals [1,2,8].

In the USA, the confusion about these terms, which were used to refer to the veterinary responsibilities of non-domestic or non-traditional animals, was addressed in the year 2000 in an intensive workshop that took place in White Oak, convened by the ACZM (American College of Zoological Medicine). As a result, there was a solid consensus to adopt a single consistently defined term to represent the wide range of activities related to non-domestic species. Eventually, the term “zoological medicine” was explicitly established by the White Oak Accords as the appropriate umbrella concept which integrates veterinary medicine and the principles of ecology and conservation applied in both natural and artificial environments [5].

Scaling up the framework of zoological medicine, there are other terms, even higher, which need clarification; these are One Medicine, conservation medicine, and the One Health concepts.

As early as 1964, Dr Schwabe coined the term “One Medicine” and proposed veterinary and human health professionals collaborate to combat zoonotic diseases [9]. Similarly, in 1996, Kock introduced the concept of conservation medicine in response to the growing concern about the adverse effects of anthropogenic environmental changes on humans, animals, and ecosystem health [10].

**Figure 1:**
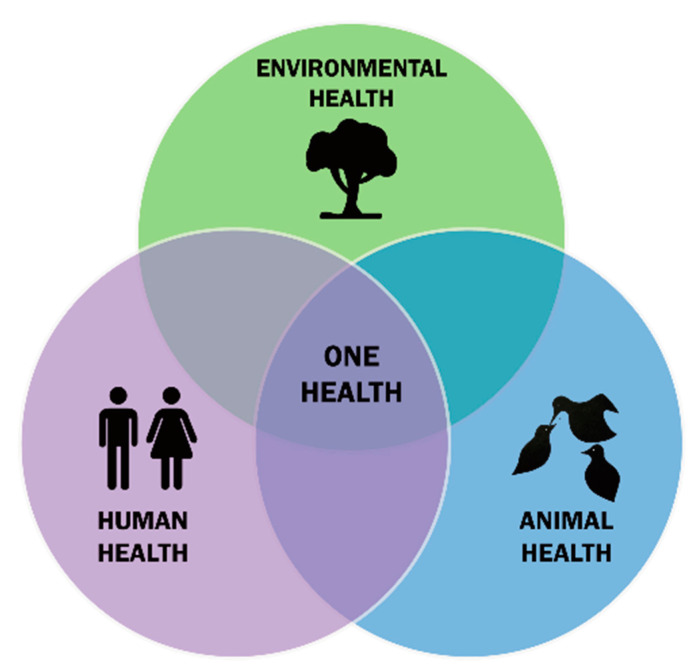
Graphical representation of One Health approach (licensed under the Creative Commons Attribution-Share Alike 4.0 International license (https://creativecommons.org/licenses/by-sa/4.0/deed.en, accessed on 26 April 2023)).

More recently, in the early 2000s, the One Health initiative emerged. The One Health approach (Figure 1) summarises a concept known for more than a century—that human, animal, and plant health are interdependent and bound to the health of the ecosystems in which they exist. The Food and Agriculture Organization of the United Nations (FAO), the World Organisation for Animal Health (OIE), the United Nations Environment Programme (UNEP), and the World Health Organization (WHO) have established a common advisory panel proposing the following definition: “One Health is an integrated, unifying approach that aims to sustainably balance and optimise the health of people, animals, and ecosystems. It recognises the health of humans, domestic and wild animals, plants, and the wider environment (including ecosystems) are closely linked and interdependent. The approach mobilises multiple sectors, disciplines, and communities at varying levels of society to work together to foster well-being and tackle threats to health and ecosystems while addressing the collective need for clean water, energy and air, safe and nutritious food, taking action on climate change, and contributing to sustainable development”.

Based on this definition, it is evident that veterinarians play a key integral role in the One Health approach because animals both impact and are impacted by people and the environment. Nevertheless, even today, human, animal, and environmental science studies are largely conducted independently [11]. Furthermore, veterinarians, being experts in animal production, welfare, and food safety and its technology, and public health under the One Health concept, are scarcely informed in environmental aspects, which would help them to understand and face the consequences of climate change [12].

### 3.2. The Practice of Zoological Medicine: From the Historical Roots to the Current New Scenarios

The role of veterinarians in dealing with wild and exotic animals is continuously increasing and changing. Progress and evolution have been different according to diverse factors. This section reviews and summarises this evolution according to the main application sectors.

#### 3.2.1. Zoological Medicine Applied to Non-Traditional Companion Animals (NTCAs) in Private Veterinary Clinics and Hospitals: From Just “Collecting Animals” to Responsible Ownership of Exotic and Non-Conventional Pets

The habit of collecting animals, particularly wild and exotic animals, is an ancient and common practice (Mesopotamia, Egypt, China, Greece, and Rome). The interest in owning wild animals was widespread among the European aristocracy between the 16th and 18th centuries because these possessions were considered prestigious and symbols of luxury and power of the nobility of those times [3,13,14].

Since these antique practices, the desire and interest in keeping wild animals in captivity have increased. During the last decades, this growth has been extended to other homes of other social classes, notably the increasing number of owners of exotic animals as new pets or non-traditional companion animals (NTCA). The European Pet Food Industry Federation (FEDIAF) estimates that NTCAs kept in captivity in European households are more than 48 million birds, more than 16 million fishes, 29 million small mammals, and 11 million reptiles and terrarium animals [15].

In the US, the same tendency has been reported. In this country, the notable increase must be highlighted, up to the highest registers in a decade, of new acquisition levels of pets other than dogs and cats, due to the motivation of COVID-19 pandemic lockdowns. Nowadays, 12.2% of US households own NTCAs as pets, up from 10.8% 4 years before, according to the 3rd edition of the Fish, Small Mammal, Herptile, and Bird Products: U.S. Market Trends and Opportunities Report [16].

Therefore, NTCAs are acquiring an increasingly important role as pets as the penetration rate into homes becomes more remarkable. Consequently, the demand for specialised veterinary care, specific knowledge, and competencies must be improved and increased in parallel. It should be remembered that the legal possession of unconventional pets is associated with attention to specialised requirements and their specific anatomical and physiological characteristics, which do not allow extrapolating procedures from the traditional medicine of dogs and cats [8].

This particularity of requirements has led different organisations, such as the Federation of Veterinarians of Europe (FVE), to request the creation of white lists of animals that can be kept in captivity as pets. That has been reinforced by the general knowledge that most emerging infectious diseases worldwide are zoonotic [17]. Some European countries that have already implemented white lists are Belgium, Croatia, Luxembourg, Greece, the Netherlands, and Norway. In addition, some states in USA and provinces in Canada have also established white lists [18].

The development of this kind of list can mean a decrease in the number of species kept in captivity and, therefore, in the number of individuals. On the other hand, it may mean a concentration of individuals in certain species which could generate greater specialisation from the point of view of veterinary care. Consequently, private veterinarians have an increasing role in supporting non-traditional pets’ welfare and health, preventing zoonosis, and acting under the One Health approach.

#### 3.2.2. Zoological Medicine Applied to Exotic and Wild Fauna in Captivity on Zoos and Parks: From “Menageries” to Bioparks

The practice of wild animal collecting, described above, depending on certain circumstances and due to various motivations, may contribute to explaining the beginnings of many zoos in different European countries [3,13].

Zoological parks have evolved from a merely exhibitionist perception to a more conservationist one. They were initially conceived as menageries [3,19,20] where wild and exotic animals were simply exposed to the public, without fulfilling any additional function than the mere entertainment and enjoyment of visitors. However, they have experienced a resounding change, and although there are still zoos with this archaic vision, many others have spent years developing education, research, conservation, and captive breeding to contribute to the survival of threatened species [8]. These zoos have become bioparks, rescue centres, or reserves that often allow visitors to see animals in more natural and less intrusive environments, replacing the barred cages with semi-open facilities based on the zoo or landscape immersion concept [3,21].

Nowadays, in most countries of the world, zoos and animal exhibitions are requested to be adapted not only to ethical but also to legal requirements to develop ex situ conservation programs, species survival programs, environmental education, and even directly, or indirectly, participation in in situ projects. Unfortunately, for some species, breeding programs and captivity maintenance are the only way to avoid extinction [22,23].

In all this, it is vital to highlight the work carried out by the veterinarians in this new concept of zoos for maintenance of fauna in captivity, not to be just a doctor dedicated to taking care of the health of captive specimens but to participate in notions of animal welfare, conservation, education, and research.

#### 3.2.3. Zoological Medicine Applied to Wildlife: From Hunting Management to Eco-Pathology and Rehabilitation Centres

Wildlife management is a complex process that should not focus exclusively on the animal but must be understood as integral management considering biological, health, sociological, and economic aspects. Consequently, the involvement of veterinary sciences is crucial [24].

Hunting and fish management programs were scarce or even non-existent until very recently. However, such management requires measures to guarantee the rational exploitation of a limited natural resource. In the past, game management focused primarily on hunting pieces and the exploitation of natural resources, and veterinarians could be hired to perform tasks such as inspecting bushmeat to ensure its quality. Over time, game management has become more focused on the conservation and sustainable management of wildlife populations [24].

Wildlife rehabilitation centres are places often managed and under the responsibility of veterinarians. They provide medical attention and care to injured or sick wild animals to recover and return them to their natural habitat. The creation of the first wildlife rehabilitation centres dates to the 1970s [25]. Since then, these facilities have spread and evolved, with governmental, non-governmental, or individual support, covering the protection of a variety of terrestrial and aquatic species. Their initiatives and actions have also evolved from providing clinical and medical care to also acting as sentinel centres evaluating and reporting on the health status of wild populations, contributing to the detection and diagnosis of key environmental threats, affecting ecosystems, and supporting the essential role of environmental education [20].

The work of veterinarians in wildlife management and rehabilitation centres provides opportunities for biomonitoring and identification of anthropogenic sources of injuries and critical threats such as environmental pollutants and new diseases [24]. The veterinarian is the only professional who combines knowledge about genetics, reproduction, zootechnics, ethology, welfare, and animal health and those authorised to handle and apply anaesthetics and euthanasia [24].

### 3.3. Social Demand and Training Needs on Zoological Medicine

WOHA (formerly OIE) set out in its recommendations the minimum competencies expected of newly licensed veterinarians to ensure the quality of national veterinary services and highlighted their essential contribution to society in ensuring the health and welfare of animals, people, and ecosystems. Furthermore, WOHA stressed the importance of high-quality veterinary training, initially and continuously during the professional exercise. This fact is consistent with an increased societal demand for skilled and trained professionals concerning biology, management, health, and conservation of wild species.

In the last decades, the emergence of zoonotic diseases on production animals derived from wildlife populations (e.g., the recent cases of MERS and SARS, monkey virus, nipha virus, etc.) has increased global awareness of the importance of zoological medicine in protecting both production livestock and public health. In this demand, zoological medicine has expanded to a wider variety of subspecialties beyond clinical care to encompass conservation, research, nutrition, reproductive physiology, molecular genetics, and others.

Veterinarians work with a unique comparative approach to conduct zoonosis research. However, in most veterinary curricula, the prevalence of domestic animal studies and the need for a system from a population-scale and ecosystem perspective pose an obstacle and create a knowledge gap in veterinary careers. With increasing funding and interest in studying the ecology of emerging infectious diseases, both human and wildlife, there is likely to be dramatically greater demand for veterinarians, ecologists, and other professionals who understand these concepts in an integrated manner [25,26]. Furthermore, knowledge of the principles of ecology and ecosystems should be acquired during pre-veterinary studies or, at least, at the beginning of the veterinary curriculum. At the graduate level, master’s degrees in preventive veterinary medicine, ecology, environmental health, or public health with an emphasis on infectious diseases should be offered to veterinarians seeking job opportunities in public health and wildlife management [27].

It is essential to take a multidisciplinary approach to deepen the study of the incidences of diseases in wild populations. Veterinary science plays a central role in this regard. Currently, the two disciplines that lead the studies of diseases in wild species are epidemiology on the side of medical sciences and ecology on the side of biology. However, given the complexity of the issue, it is necessary to promote the formation of multidisciplinary teams to address the health aspects of fauna, species conservation, animal production, ecosystem health, and public health [28].

Therefore, conservation medicine and zoological medicine face challenges in the academic field, where they must make their way into a space theoretically already occupied by different disciplines and subdisciplines. Ecosystem health and conservation medicine agree that an approach to health and treatment should be part of the basic training received by health sciences students in general and among veterinarians [29].

It is time for veterinarians to create formal mechanisms within academic institutions to promote and increase enthusiasm for wildlife and ecosystem health. This transdisciplinary view will encourage a more global, generational, and preventive approach to health care. Veterinary faculties have a unique opportunity to collaborate in teaching these complexities, as veterinary schools employ specialists working from the ecosystem and population levels to the environmental and molecular. Thus, progress has been made in expanding veterinary curricula to provide basic critical knowledge and skills needed to provide medical care to captive non-domestic or non-traditional species. Furthermore, most recent veterinary curriculum revisions have already been considered, including studying animal populations and their environmental interactions [30].

### 3.4. Educational Programs and Veterinary Perception of Professional Skill and Competence in Zoological Medicine

Europe has a long and distinguished history in veterinary science and education, and it was here that the first professional investigations of pathological conditions in zoo animals were carried out. However, despite the increasing number of veterinarians working with wildlife, education in zoological and wildlife medicine has only recently and partially been included as part of formal veterinary training at the undergraduate level.

Due to the large number and diversity of nation-states in Europe, current educational opportunities in zoological medicine vary widely across Europe, both in availability and composition and at both undergraduate and post-graduate levels. The need to establish agreed-upon standards in education across Europe and to encourage the mobility of students and teaching staff is reflected in international agreements such as the Bologna Process and the ERASMUS-Socrates program and is likely to help reduce these differences and have a positive effect on the role of zoo and wildlife medicine in veterinary education [31,32].

Different European studies have tried to assess the degree of competence, educational programs, and availability of specialised veterinarians in zoological medicine and exotic and non-traditional companion animals. As early as 1994, Zwart investigated undergraduate training in zoological medicine in 27 European veterinary faculties, revealing that although there was a growing interest in exotic animal medicine, exotic animal diseases were taught separately as electives [31].

During the spring–summer of 2005, Mazet and colleagues conducted a survey designed to identify the educational and training needs of people entering the fields of wildlife medicine and ecosystem health. Data revealed that only some wildlife veterinarians believed that the training they received in veterinary school adequately prepared them to acquire and succeed in their field. Instead, wildlife veterinarians and their employers rated mentorship with an experienced veterinarian, leadership and communication training, wildlife health courses and internships, and additional formal training beyond the veterinary degree as necessary in preparing for success. In addition, survey responses from employers, wildlife veterinarians, and job seekers demonstrate that understanding and maintaining ecosystem health is a crucial component of the wildlife veterinarian’s job description, as it is critical to protecting animal health, including human health [30].

A survey conducted in Ireland in 2020 by Goins and colleagues revealed a third of respondents had an exotic pet, and 50% of them had requested a veterinary consultation in the previous year. Most of them found barriers to accessing veterinary services, highlighting the perception of a lack of species-specific competence in the provision of veterinary services [33].

In parallel, another survey conducted by the same authors showed that the prevalence of veterinary services for exotic pets in Ireland was present in 82% of small and mixed animal clinics. Furthermore, more than four out of five veterinary professionals in small or mixed animal practices surveyed were willing to treat exotic pets. This fact contradicts the views expressed by respondents in the other survey, which indicated a limited availability of veterinary services. This contradiction could be explained because veterinarians offer a first aid or primary care approach. At the same time, pet owners may seek veterinarians who specialise and have post-graduate training in their pet’s species [15].

Ostovic and collaborators surveyed in the academic year 2019–2020 veterinary students from Zagreb (Croatia) from different courses related to the veterinary medicine of reptiles. The study also revealed the need to invest in efforts to update the veterinary curriculum to introduce additional education for future Croatian veterinarians in reptile medicine since, in addition to clinical practice, this problem has implications for the health and safety of humans and other animals, as well as for the protection of the environment [34].

Recently, a study by Roopnarine and collaborators aimed to explore the faculty perceptions in medical, veterinary, and public health programs on the need, opportunities, and challenges of developing the concept of One Health in curricula, revealing that faculty perceived education in One Health as crucial to preparing veterinary students for collaborative practice. Successful One Health development is vital to prepare students for future threats to global health and promote a culture of shared learning [35].

In North America, advances in zoological medicine education were notably facilitated by the deliberations and recommendations of the White Oak Accords of 2000. A group of veterinarians teaching at universities in the United States created a steering committee and organised a workshop under the auspices of the ACZM to study and implement improvements in North American veterinary curricula in birds, reptiles, small pet mammals, non-traditional companions, aquatic and zoos animals, wildlife medicine, and environmental health and conservation. The committee and the ACZM recognised that the most significant demand was in providing medical care to companion animals due to the large proportion of graduates who wished to pursue careers in small animal clinical practice. Five years after these agreements, a review of curricular opportunities at USA and Canadian veterinary schools showed that progress had been made in implementing those recommendations. However, there is still room for improvement [7].

A survey conducted in 2000 by Stoskopf et al. revealed that 21 of the 31 North American veterinary colleges included aspects of zoological medicine within the curriculum and all offered educational opportunities in zoological medicine. The same authors recommended to the AVMA (American Veterinary Medical Association) that all veterinary schools train students to work in small animal medicine by acquiring proficiency in birds, reptiles, small mammals, and fish and that, at a minimum, they should teach clinical skills, comparative anatomy, physiology, and behaviour.

Although specialisation in zoological medicine is well-established in the UK, USA, and Australia, the number of residency programs in zoological medicine currently needs to be increased to meet the needs of this field. Therefore, veterinary schools, zoos/aquariums, or alliances between these institutions must establish additional training opportunities for veterinarians in this discipline [1,31,35].

Other geographical contexts have also assessed the implementation of zoological medicine and the work with exotic animals and NTCAs. For instance, a study conducted in Guatemala in 2018 revealed the lack of confidence that most veterinarians surveyed have when they must work with wild and exotic animals; even though they indicated that 92.3% were directly attended to and treated, only 5.6% were referred to another clinic, and 2.1% were not attended [36].

In Turkey, a study was also carried out on the perception of the training received for the clinical practice of exotic animals at the Istanbul Faculty of Veterinary Medicine. A total of 90% of the veterinarians felt they needed additional training at the university. More than 65% thought they had adequate knowledge about managing, transmitting, preventing, diagnosing, and treating avian diseases but did not have that knowledge regarding turtles, other reptiles, and fish. The frequency of care of exotic pets significantly affected veterinarians’ confidence in treating them. Knowledge and confidence in the diagnosis and treatment of exotic pets were considerably lower than for dogs and cats [37].

To contribute to and update these studies, we launched a survey to evaluate some key parameters of the implementation of zoological medicine in Spain. The survey was anonymous and distributed online among professional veterinary associations between December 2022 and January 2023. A number of 120 professionals answered it. The results showed that 89.3% of respondents work in the small animal practice, similar to the data reported by Goins and Lepe-Lopez in Ireland and Guatemala, respectively.

**Figure 2:**
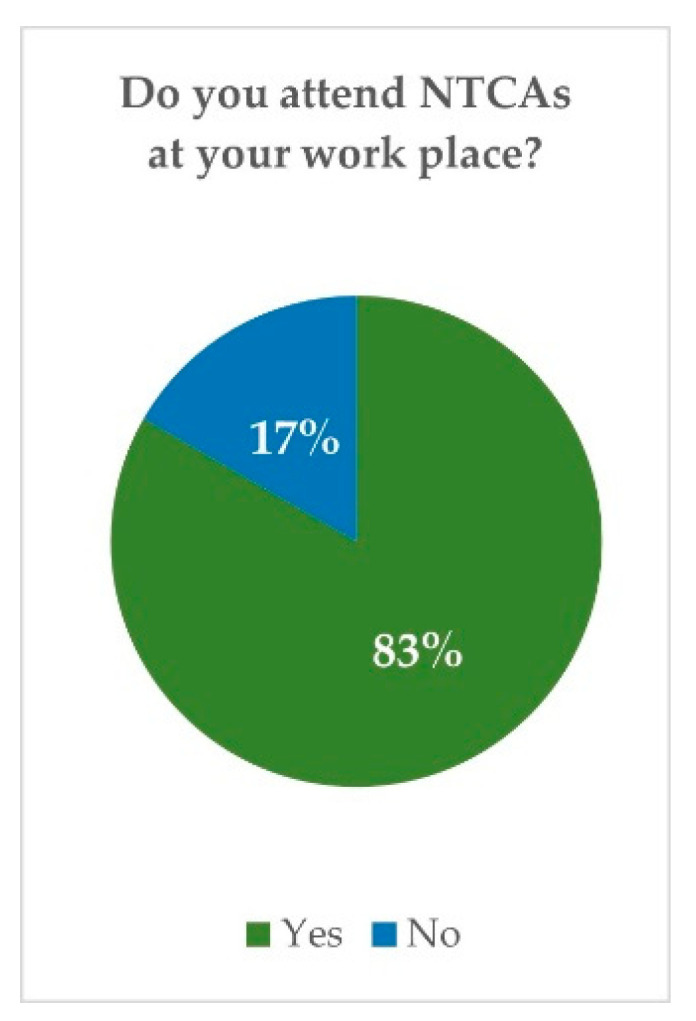
Spanish survey: percentage of veterinarians who attend NTCAs.

**Figure 3:**
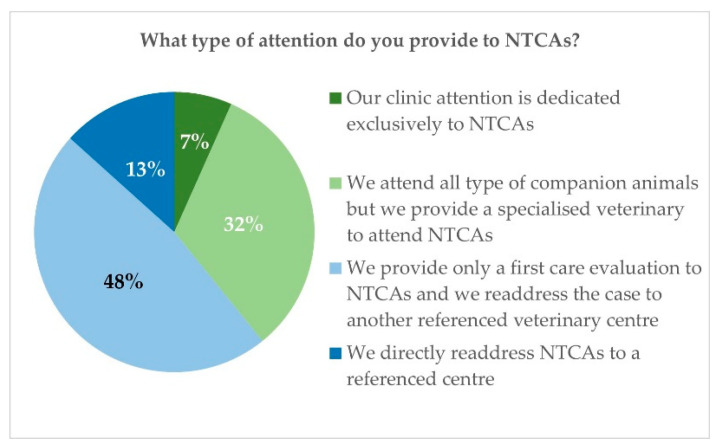
Spanish survey: type of attention applied to NTCAs.

Among these veterinarians in Spain, 83.3% confirmed they receive non-traditional companion animals in their consultancies (Figure 2). Most of them (47.5%) said to provide first aid care as an essential service and readdress the case to a reference centre specialised in medical care for these animals. Only 6.7% declared to attend exclusively exotic or non-traditional animals and 32.5% to have a specific member of the staff who has the knowledge, competencies, and skills to attend non-traditional companion animals in their clinics (Figure 3).

**Figure 4:**
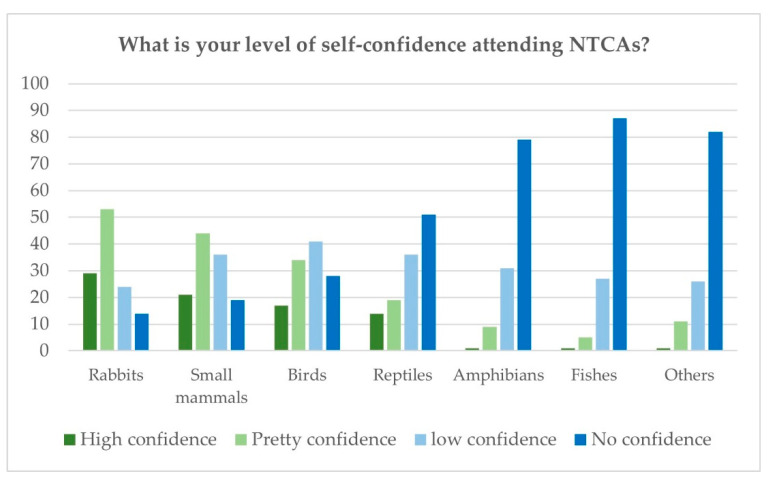
Spanish survey: level of self-confidence attending different NTCAs.

The only taxa where respondents said they mostly felt confident were rabbits (68.3%) and small mammals (54.2%). They felt little or no confidence in dealing with other birds (57.5%), reptiles (72.5%), amphibians (91.7%), fish (95%), or other non-traditional companion animals (90%) (Figure 4).

**Figure 5:**
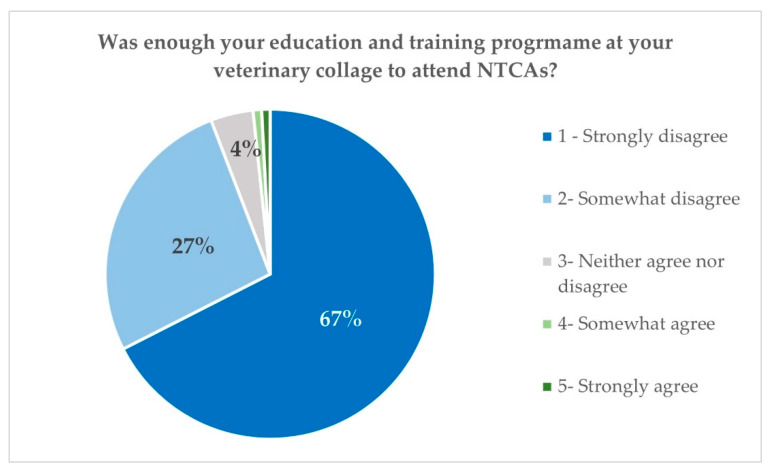
Spanish survey: level of specialised education received at the veterinary college.

**Figure 6:**
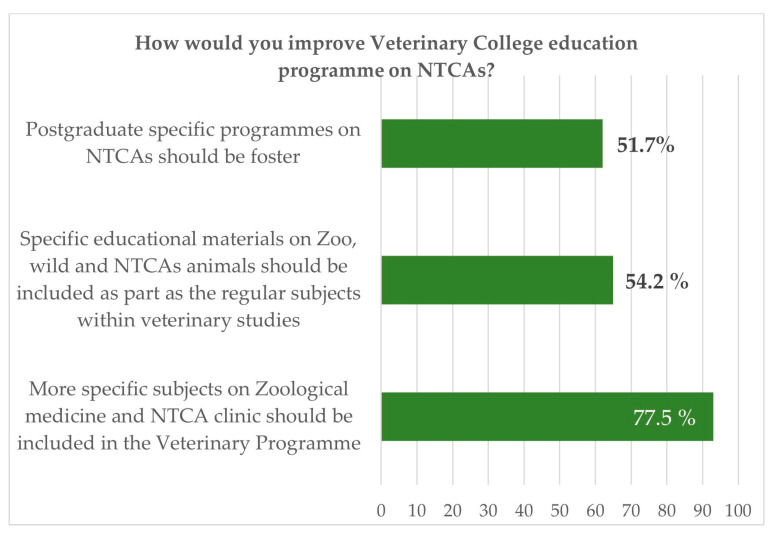
Spanish survey: suggested measures to improve NTAC specialised training. More than one option could be selected by respondents.

When they were asked if the education received at the university was enough, on a Likert scale from 1 to 5, 67.2% considered it as null (1), 31.9% medium (2–4), and only 1 person estimated it as enough (Figure 5). The average was established at 1.17. Further, 77.5% of the respondents declared the need to increase the number of specific subjects of zoological medicine and NTCAs in the Veterinary curricula (Figure 6). These results are in coincidence with those reported by Mazet et al., 2006, from veterinarians’ answers in North America, Mexico, the Philippines, Australia, New Zealand, France, Brazil, South Africa, and Scotland (UK). All these surveys and studies imply the need for pre-and post-graduate veterinary education to support the veterinary community in providing services to NTCA pet owners and to wildlife care.

The curriculum should address the enormous diversity of wild animals, from amphibians to marine mammals and from insects to fish, seen in a zoo, aquarium, park, or veterinary practice, including an understanding of the local and global implications of emerging infectious diseases and preventive medicine in zoological collections. Training related to veterinary ethics and animal welfare science applied to zoos and wildlife should also be required [38].

## 4. Conclusions

Every day the role of veterinarians is more relevant and necessary in supporting the welfare and health of non-traditional companion animals and wildlife animals, and also human and environmental health. The importance of the One Health/One World concept and its social impact is increasing, especially accompanied by the notoriety of new emerging and reemerging zoonoses.

Within this framework, zoological medicine should be the appropriate and agreed-upon term in the academic language concerning the veterinary medicine of pets, wild, or zoo species, excluding traditional domestic animals, and integrating the principles of ecology and conservation, applied to both natural and artificial environments. Zoological medicine has evolved from its historical roots to the current new scenarios in a different way depending on the fields of work. These fields, even sharing focus and objectives, have their particularities and demand specific lines of action.

In this sense, private veterinarians’ role in supporting non-traditional pets’ welfare and health is increasing as these animals’ penetration rate into homes becomes more notable. At the same time, veterinarians can contribute to the prevention of zoonosis under the One Health approach.

Zoological medicine applied to exotic and wild fauna in captivity in zoos and parks has evolved from the old conceptions of menageries to centres of conservation, education, and research, and the veterinarians working in these places should not focus only on the health of captive specimens. In the same way, the work of veterinarians in wildlife management and rehabilitation centres should contribute to recovering injured animals and identifying and monitoring environmental threats.

All this implies current and future challenges for the veterinary profession, at all international levels, that can only be addressed with greater and better attention from multiple perspectives, especially the education and training of professionals to improve and specialise in their professional scope of services.

The present work reinforces that new approaches are needed in training, both undergraduate and post-graduate, in all veterinary schools throughout the world and in Spain and the rest of Europe. This situation should be more comprehensive than including certain content in core subjects or elective courses, but instead, more compulsory courses and truly accredited training itineraries should be generated. Different studies and surveys across the world, including our survey, support this position.

## Data Availability

All data are available on request.
